# An attenuated *Mycobacterium tuberculosis* clinical strain with a defect in ESX-1 secretion induces minimal host immune responses and pathology

**DOI:** 10.1038/srep46666

**Published:** 2017-04-24

**Authors:** Helena Strand Clemmensen, Niels Peter Hell Knudsen, Erik Michael Rasmussen, Jessica Winkler, Ida Rosenkrands, Ahmad Ahmad, Troels Lillebaek, David R. Sherman, Peter Lawætz Andersen, Claus Aagaard

**Affiliations:** 1 Department of Infectious Disease Immunology, Statens Serum Institut, DK-2300, Copenhagen, Denmark; 2 International Reference Laboratory of Mycobacteriology, Statens Serum Institut, DK-2300, Copenhagen, Denmark; 3 Center for Infectious Disease Research, Seattle, Washington, 98109, USA

## Abstract

Although *Mycobacterium tuberculosis (M.tb*) DK9897 is an attenuated strain, it was isolated from a patient with extrapulmonary tuberculosis and vaccination with a subunit vaccine (H56) induced poor protection against it. Both attenuation and lack of protection are because *M.tb* DK9897 cannot secrete the EsxA virulence factor nor induce a host response against it. Genome sequencing identified a frameshift mutation in the *eccCa1* gene. Since the encoded EccCa1 protein provides energy for ESX-1 secretion, it suggested a defect in the ESX-1 type VII secretion system. Genetic complementation with a plasmid carrying the *M.tb* H37Rv sequence of *eccCa1-eccCb1-pe35* re-established EsxA secretion, host specific EsxA T-cell responses, and increased strain virulence. The ESX-1 secretion defect prevents several virulence factors from being functional during infection and therefore attenuates *M.tb*. It precludes specific T-cell responses against strong antigens and we found very little *in vivo* cytokine production, gross pathology or granuloma formation in lungs from *M.tb* DK9897 infected animals. This coincides with *M.tb* DK9897 being unable to disrupt the phagosome membrane and make contact to the cytosol.

The facultative intracellular pathogen *Mycobacterium tuberculosis (M.tb*) is one of the most devastating human pathogens in the world. Although the incidence rate of tuberculosis (TB) is on a steady decline, TB remains one of the major public health problems, with 9 million new cases and the deaths of around 1.5 million people each year^1^.

Pathogenic mycobacteria have the ability to resist killing by phagocytic cells of the host immune system. Phagocytic cells degrade invading microbes by engulfment inside a vacuole or phagosome that progressively acidifies and accumulates hydrolytic properties. *Mycobacterium marinum* and *Mycobacterium avium* are opportunistic pathogens that can survive and proliferate in host cells because they are capable of blocking accumulation of the vacuolar H^+^-ATPase on the mycobacterial vacuole and prevent delivery of the lysosomal protease cathepsin D^2^,^3^,^4^. *M.tb* inhibits phagosomal maturation and the endosomal-lysosomal degradation pathway and can, therefore, manipulate antigen presentation^5^. Very recent data have shown that EsxH (TB10.4) and EsxL, substrates of the ESX-3 and ESX-5 type VII secretion systems respectively, are part of this control. EsxH prevents the ability of antigen presenting cells to activate CD4 T cells by inhibiting the endosomal sorting complex required for transport (ESCRT) machinery and EsxL inhibits major histocompatibility complex class II (MHC-II) expression by enhancing the methylation of a transactivator loci^6^,^7^. All these defense mechanisms reduce epitope presentation on the surface of infected cells and subsequently affect the adaptive immune response in terms of delayed recruitment of T cells to the site of infection and suboptimal T cell activation of infected cells^8^,^9^. In addition, virulent *M.tb* also exploits the ESX-1 type VII secretion system to secrete virulence factors that are involved in survival and spreading of the pathogen via interactions with the host cells^10^,^11^.

Comparative analysis of genomes from attenuated *M. bovis* BCG strains and pathogenic mycobacterial species identified the main chromosomal ESX-1 locus, containing region of difference 1 (RD1) genes, and showed that this region encodes the immunodominant T cell antigens EsxA (ESAT-6) and EsxB (CFP-10)^12^,^13^,^14^. RD1 gene complementation not only re-established the expression and secretion of EsxA and EsxB but also increased the virulence of *M. bovis* BCG^15^. Deleting single genes in the *M.tb* ESX-1 locus, encoding core components of the ESX-1 apparatus, blocked EsxA and EsxB secretion and attenuated the bacillus in cellular and animal models of infection^16^.

After synthesis, EsxA and EsxB form a heterodimer inside the mycobacterial cytoplasm. EsxB has a dual function as a chaperone and secretion partner, holding the sequence needed for secretion of the dimer via ESX-1. Once secreted, the heterodimer dissociates at low pH in the acidic environment of the phagosome. EsxA has been reported to be involved in numerous biological processes relevant for virulence including; initiation of granuloma formation^17^, phagosome maturation^18^,^19^, apoptosis through caspase activation^20^ and induction of membrane damage and phagosomal disruption^21^. Two most recent studies demonstrate that EsxA is not directly responsible for membrane lysis, rather this activity is attributed to ESX-1 in concert with phthiocerol dimycocerosates (DIMs) and is contact dependent, which results in gross membrane disruptions rather than pore formation^22^,^23^. ESX-1 has also been shown to be involved in host cell immune modulation^24^,^25^.

The isolation of an *M.tb* strain unable to secrete EsxA from a Danish patient with extrapulmonary TB was unexpected because of its importance as a virulence factor for *M.tb*. Here we report on the initial characterization of this clinical isolate and the responses it induces in the host during infection.

## Results

### *M.tb* DK9897 belongs to a lineage with few members

Since *M.tb* strains from different lineages can induce variable host responses in macrophages, cell lines and mouse models^26^,^27^,^28^ the genetic diversity among lineages could potentially influence the protective efficacy of TB vaccines. We, therefore, set out to test the ability of the H56 vaccine^29^ to protect against aerosol infection with clinical *M.tb* isolates. H56 is a fusion protein of the *M.tb* proteins Ag85B, EsxA, and Rv2660c.

**Figure 1:**
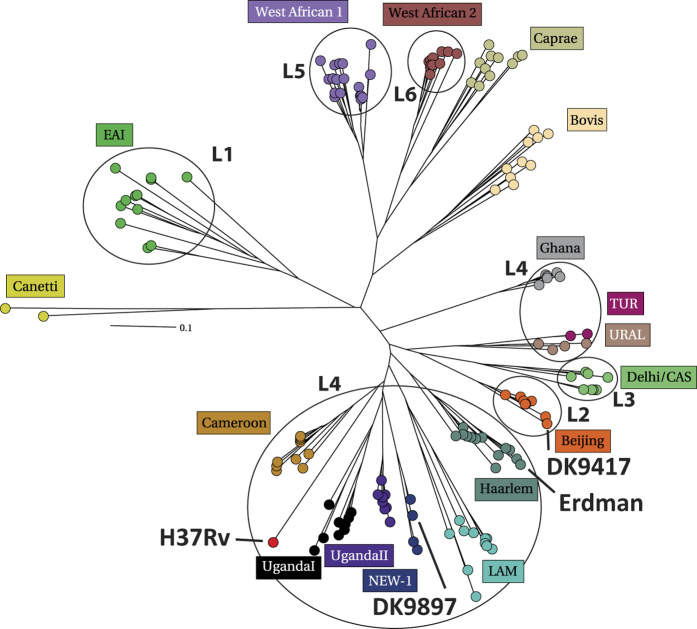
The unique *M.tb* DK9897 isolate is related to laboratory-adapted strains. Phylogenetic distribution of *M.tb* isolates based on MIRU-VNTR typing. A radial tree was constructed based on the neighbor-joining algorithm and categorical distance coefficient using the 24-locus MIRU-VNTR typing data for DK9897 and all isolates available in the MIRU-VNTR+ database as input.

The *M.tb* DK9897 isolate was one of six clinical isolates selected from the *M.tb* strain collection at the International Reference Laboratory of Mycobacteriology harboring >ten thousands of clinical isolates cultured from individuals infected with mycobacteria. In our selection, we prioritized lineage coverage and sequence diversity but for safety reasons, we only included strains that were susceptible to standard anti-tuberculous treatment. *M.tb* DK9897 was originally isolated in February of 1998 from the cervical pus of a 92-year-old woman with tuberculous lymphadenitis. The isolate was susceptible to isoniazid, rifampicin, ethambutol, pyrazinamide and streptomycin. To investigate if *M.tb* DK9897 was part of a larger subgroup of mycobacterial isolates we genotyped the *M.tb* DK9897 strain along with the laboratory-adapted strains *M.tb* Erdman and *M.tb* H37Rv and an isolate belonging to the large Beijing family, *M.tb* DK9417. One quick and reliable marker commonly used for *M.tb* genotyping is the mycobacterial interspersed repetitive units (MIRU), located in variable number tandem repeats (VNTR) found at multiple loci scattered throughout the genome. The MIRU-VNTR genotyping data (Supplementary Table S1) was uploaded to the MIRU-VNTRplus database and a phylogenetical analysis was performed using a neighbor-joining algorithm and categorical distance coefficient using our four 24-locus MIRU-VNTR typing data and all isolates available in the MIRU-VNTR+ database as input. The results show that DK9897 does not belong to any of the established lineages but is a member of a new lineage with very few members that cluster between the *M.tb* Erdman and *M.tb* H37Rv (Fig. 1).

### The H56 subunit vaccine induced poor protection against *M.tb* DK9897

**Figure 2:**
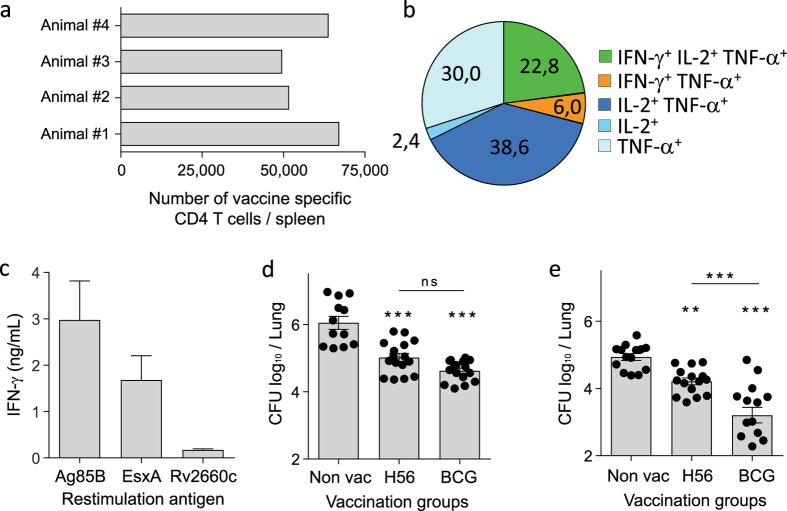
Strong H56 vaccine-take only induces moderate protection against the *M.tb* DK9897 strain. (**a**) The number of vaccine-specific CD4 T cells was measured by flow cytometry in splenocytes 3 weeks after third vaccination. CD4 T cells that after re-stimulation with a cocktail of H56 single proteins (Ag85B + EsxA + Rv2660c) produced at least one of the cytokines, TNF-α, IFN-γ, IL-2 or IL-17 were scored as vaccine specific. Results from four individual mice are shown. (**b**) The same spleen cells were used to investigate the polyfunctionality of the H56 specific T cells by measuring the frequencies of the seven possible combinations of TNF-α, IFN-γ and/or IL-2 producing T cells after stimulation with H56 proteins. The pie shows an average of the four animals. (**c**) The ability of CD4 T cells isolated from vaccinated mice (n = 4) to recognize the three single proteins in the H56 fusion protein was measured as IFN-γ release after restimulation with either Ag85B, EsxA or Rv2660c protein. (**d**,**e**) Bacterial load in individual lungs six weeks after aerosol infection with *M.tb* Erdman (**d**) or *M.tb* DK9897 (**e**), respectively. Measured by plating serial dilutions of lung homogenate and counting the number of mycobacteria after incubation. Combined results from two independent experiments are shown. Mean values and SEM’s are indicated. Statistical analysis, One-way ANOVA and Tukey’s multiple comparison tests. ***p < 0.001, **p < 0.01, ns = non-significant.

Groups of mice were vaccinated with either H56 fusion protein in CAF01 adjuvant or BCG. Three weeks after the third H56 vaccination, the vaccine-specific T cell responses were investigated by flow cytometry in splenocytes. CD4 T cells that after stimulation with a pool of the three single antigens in H56 produced at least one of the cytokines IFN-γ, tumor necrosis factor-alpha (TNF-α or IL-2 were taken as being vaccine specific. Overall, the H56 vaccine-induced immune responses, in terms of T cell polyfunctionality and frequency of vaccine-specific cells, was at a similar level to what we have seen before^30^. On average 58.073 ( +/− 8704) of the spleen CD4 T cells recognized the vaccine (Fig. 2a). More than 60% of these were IL-2 producing T cells with high proliferative capacity and potential to become memory cells (Fig. 2b). The strongest T cell response was against Ag85B followed by EsxA and then a weak recognition of Rv2660c (Fig. 2c). Thus, we had a robust high-quality T cell response in the H56 vaccinated animals that previously have resulted in efficient protection against *M.tb*^30^. The expression of the Rv2660c gene has been questioned since no Rv2660c gene expression was found in cultures starved for nutrition’s nor in mice infected with *M.tb* for up to 31 days with *M.tb*, instead strong expression of a small RNA on the opposite strand was found^31^. Yet, in two studies investigating human immune responses, more than 60% of persons with LTBI had T cell responses against Rv2660c and half of the TB patients responded specifically against a peptide pool covering the Rv2660c sequence^32^,^33^. Furthermore, immune mice and non-human primates have clear recall responses to Rv2660c emphasizing the availability of this antigen for recognition by primed T cells^30^,^34^. Thus, it is possible that the Rv2660c gene expression is tightly controlled and transcription only takes place under certain conditions such as e.g. immune stress. Six weeks after the third vaccination all mice were either aerosol challenged with *M.tb* Erdman or *M.tb* DK9897. The challenge dose was approximately 100 viable bacilli per animal for all infections. To compare *M.tb* strain virulence and vaccine protective efficacy the number of mycobacteria was determined in individual lungs from *M.tb* Erdman (Fig. 2d) and *M.tb* DK9897 infected (Fig. 2e) animals. Combined results from two independent experiments are shown for both strains (n = 12–16/grp). Within the six-week infection period, *M.tb* Erdman reaches an average of approximately one million bacteria per lung (Log_10_ = 6.05 +/− 0.19 SEM) whereas *M.tb* DK9897 grew to a level approximately 10-fold lower with less than 100.000 colony forming bacteria (CFU) per lung (Log_10_ = 4.93 +/− 0.10 SEM) in non-vaccinated animals. In terms of protection, *M.bovis* BCG and H56 both protected quite effectively against the *M.tb* Erdman challenge with no statistical difference in protection. However, in *M.tb* DK9897 challenged animals H56 vaccination only reduced the CFU load 5-fold compared to non-vaccinated animals whereas the *M.bovis* BCG vaccinated group had a 50-fold reduction in bacterial number, and a significant difference (p < 0.001) compared to H56 vaccinated.

### No EsxA secretion during *M.tb* DK9897 infection

**Figure 3:**
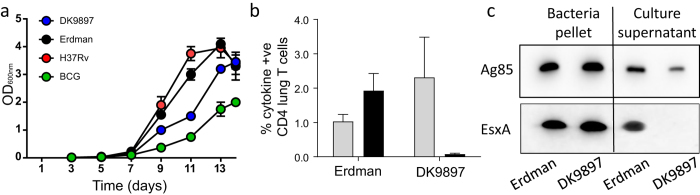
EsxA is expressed but not secreted in the *M.tb* DK9897 isolate and not recognized by T cells after infection. (**a**) *In vitro* growth of mycobacterial strains H37Rv, Erdman, BCG, and DK9897. Exponentially growing bacteria cultures (OD_600_~0.3) were diluted to a concentration of 2 × 10^5^ CFU/mL in 7H9 medium and growth was measured for up to 14 days. Data from one of two independent experiments. (**b**) Six weeks after infection with either *M.tb* strain Erdman or DK9897, cells were isolated from individual lungs and stimulated *in vitro* with either Ag85B (gray bars) or EsxA (black bars) protein. The percentage of CD4 T cells producing at least one of the cytokines, IFN-γ, TNF-α, IL-2 or IL-17 in response to stimulation was measured by flow cytometry for each group (n = 4). Mean values and SEM’s are shown. (**c**) The presence of EsxA and Ag85B proteins in bacteria pellet and culture supernatant was determined by Western blotting using *in vitro* cultures of *M.tb* Erdman and *M.tb* DK9897 and protein specific antibodies. For clarity the blots are cropped, full-length blots can be seen in Supplementary Figure S2.

*M.tb* DK9897 only multiplied from 100 to approximately 100.000 bacteria in non-vaccinated animals during the six-week infection period – 10-fold less than *M.tb* Erdman. To investigate if the difference was because of a general longer doubling time relative to *M.tb* Erdman or it was specific for *in vivo* growth conditions we compared *in vitro* growth of the two strains, *M.tb* H37Rv and *M.bovis* BCG in rich medium. During the exponential phase, the virulent strains *M.tb* Erdman and *M.tb* H37Rv grow faster than *M.tb* DK9897 that again grows faster than the attenuated *M. bovis* BCG (Fig. 3a). Thus, also under *in vitro* conditions without nutrient limitations, immune pressure and an intracellular localization, we found a reduced growth rate for *M.tb* DK9897 compared to the more virulent *M.tb* strains. To understand why the H56 vaccine protects poorly against an attenuated *M.tb* strain we examined the vaccine-specific CD4 T cell response in lungs of infected animals. After six weeks of infection, a significant portion of the CD4 T cells (1 to 2%) recognized Ag85B regardless if the animals were infected with one or the other strain. As expected, T cells from *M.tb* Erdman-infected animals recognized EsxA (approximately 2% of the CD4 T cells) but we found no T cell recognition of EsxA in *M.tb* strain DK9897 infected animals (Fig. 3b). Even in animals vaccinated prior to *M.tb* DK9897 infection, there was barely a detectable CD4 T cell response specific for EsxA in the lung (Supplementary Figure S1). To investigate if *M.tb* DK9897 expresses and secretes the EsxA protein we grew the bacteria in parallel with *M.tb* Erdman in liquid cultures. During the exponential phase, we separated the cultures into bacteria pellet and culture supernatant and visualized the presence of the EsxA and Ag85B proteins in each fraction by western blot using specific antibodies (Fig. 3c). We found Ag85B in pellet and supernatant from both cultures confirming that both strains can express and secrete Ag85B correctly. In contrast, we only found EsxA in the pellet from both cultures. EsxA was undetectable in the culture supernatant from the *M.tb* DK9897 culture. Thus, both strains express EsxA, but *M.tb* DK9897 is unable to secrete the protein.

### A frameshift in the EccC1 protein

Whole genome sequencing of the *M.tb* DK9897 strain (GenBank ID: CP018778) revealed almost 1200 mutations compared to the published *M.tb* H37Rv sequence^35^. The large majorities of these changes are either silent or influence only a single amino acid in a protein. However, there were also a number of changes with more radical effects such as frameshift mutations, insertions, deletions and stop codon loss or gain (Table 1). Given that the ESX-1 system facilitates EsxA secretion, we specifically looked for significant changes in proteins identified as being part of this secretion system. The only ESX-1 related protein that differed from the H37Rv sequence was the *eccCa1* (Rv3870) gene (Supplementary Table S2). In *M.tb* DK9897, there is a single adenosine insertion at nucleotide position 1490 leading to a frameshift in the downstream part of the open reading frame (ORF). The frameshift adds 12 new amino acids and a stop codon to the ORF after the insertion. Thus, *M.tb* DK9897 potentially expresses a truncated EccCa1 protein lacking the 305 C-terminal amino acids (Supplementary Figure S3). Using polyclonal antibodies raised against a 15mer peptide or a 101 amino acid fragment in EccCa1 we were not been able to get a positive signal with these antibodies in protein samples from *M.tb* DK9897 whereas both antibodies detected a protein with the correct size in *M.tb* Erdman samples. Thus, a truncated EccCa1 protein is likely either unstable or expressed in very low amounts. We cannot eliminate the possibility that the nucleotide insertion happened during culturing of the strain but we consider this unlikely since we kept the number of *M.tb* DK9897 passages at a minimum. We grew *M.tb* DK9897 once for initial typing and storage and subsequently used this stock to inoculate two separate cultures for sequencing which both gave identical results.

### ESX-1 polymorphisms in clinical isolates

In order to acquire a better understanding of how common mutations that are significantly enough to influence ESX-1 secretion are among clinical *M.tb* isolates we searched for polymorphisms of twelve genes known to be associated with and critical for ESX-1 secretion. We used an alignment-based search among all fully sequenced genomes of clinical isolates of the *M.tb* complex deposited in the NCBI database to evaluate the integrity of the orthologues genes in 143 genomes. Our search revealed that the twelve ESX-1 associated genes, in general, have an overall high degree of sequence conservation, with more than 99% nucleotides identical to their orthologues from the reference strain *M.tb* H37Rv. When aligned to the corresponding genes in *M.tb* H37Rv and evaluated for frameshift mutations (in/del) and stop codon loss/gain 18% (26 genomes) had one or up to six critical mutations in one of more of the investigated genes that were likely to prevent or diminish ESX-1 secretion (Supplementary Table S3).

### Genetic complementation of *eccCa1* restores EsxA secretion and increases virulence

**Figure 4:**
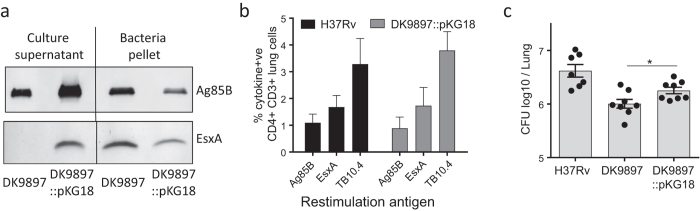
Gene complementation of *eccCa1 (Rv3870*) restores EsxA secretion, EsxA specific T cell responses and increases virulence. (**a**) Western blot comparing secretion of EsxA and Ag85B in culture filtrate and bacterial pellet from *in vitro* grown cultures of *M.tb* DK9897 and *M.tb* DK9897 complemented with the Rv3870-72 gene cassette (DK9897::pKG18). For clarity the blots are cropped, full-length blots can be seen in Supplementary Figure S2. Lung cells isolated from mice infected for six weeks with H37Rv or the complemented DK9897 strain (DK9897::pKG18) were stimulated with either Ag85B, EsxH (TB10.4) or EsxA protein. The following day the percentage of CD4+ CD3+ double-positive cells producing at least one of the cytokines TNF-α, IFN-γ, IL-2 or IL-17 in response to antigen stimulation was determined for individual mice (n = 4) by flow cytometry. (**c**) Individual lungs from mice infected with H37Rv, DK9897 or DK9897::pKG18 for six weeks were homogenized and plated in serial dilutions. After two weeks incubation, the number of mycobacteria was determined. Mean and SEMs are indicated, p = 0.03, two-tailed t-test.

To confirm that the lack of EsxA secretion was because of a non-functional eccCa1 protein we complemented the *M.tb* DK9897 with the pKG18 plasmid expressing the *M.tb* H37Rv *eccCa1-eccCb1-pe35* genes. Western blot using EsxA and Ag85B specific antibodies and culture filtrate and bacterial pellet from *in vitro* grown *M.tb* DK9897 and *M.tb* DK9897::pKG18 confirmed that complemented *M.tb* DK9897::pKG18 could express and secrete EsxA (Fig. 4a). Animals infected with either *M.tb* H37Rv or *M.tb* DK9897::pKG18 had a similar number and relative frequency of lung CD4 T cells recognizing the mycobacterial antigens Ag85B, TB10.4, and EsxA (Fig. 4b). We compared the virulence of the complemented *M.tb* DK9897::pKG18 and *M.tb* DK9897 by enumerating the number of bacteria in the lungs of mice infected for six weeks. Within this period, *M.tb* DK9897 grew to a log_10_ CFU value of 6.00 +/− 0.08 (SEM) whereas this was increased to log_10_ 6.25 +/− 0.06 in the complemented strain. The statistical comparison showed this increase to be significant (Fig. 4c). Thus, complementation of *M.tb* DK9897 with the *M.tb* H37Rv *eccCa1-eccCb1-pe35* gene cassette restores EsxA secretion into the surroundings and this leads to the generation of host specific T cell responses against EsxA and an increase in the virulence of the strain *in vivo*.

### Weak host response during *M.tb* DK9897 infection and little pathology

**Figure 5:**
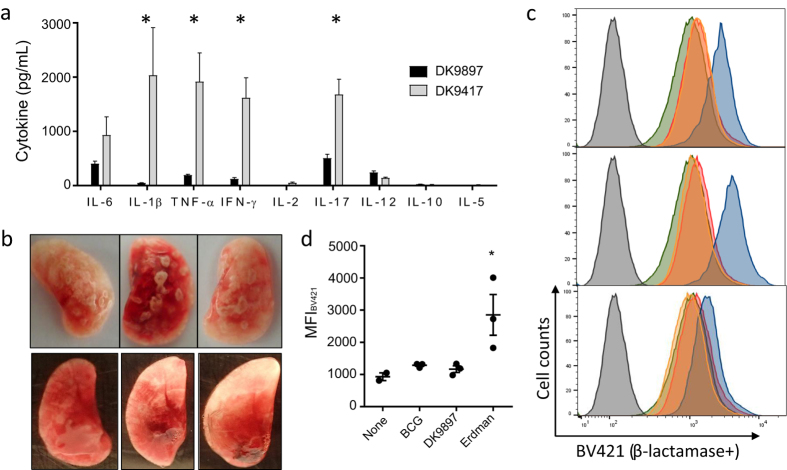
*M.tb* DK9897 infection induces very little host immune response and gross pathology. (**a**) Using a multiplex assay the *in vivo* levels of nine cytokines were measured in supernatants from homogenized lungs taken six weeks after infection with *M.tb* DK9897 or *M.tb* DK9417 (n = 4 per infection group). Mean and SEMs are indicated, *p < 0.05, two-tailed t-test. (**b**) Photographs of half-lungs from mice infected for six weeks with *M.tb* Erdman (top) or *M.tb* DK9897 (bottom, darker background), n = 3 for each. (**c**) Phagosomal membrane disruption was measured with a CCF-4 based FRET assay using flow cytometry. THP-1 cells were differentiated with PMA for 72 h and infected with 5 MOI of either *M.tb* Erdman (blue), *M.tb* DK9897 (orange) or BCG (red) for 2 h (n = 3). Four days post infection cells were stained with CCF-4 for 2 h and fluorescence was measured using 405 nm as excitation laser and 450/50 nm (blue) and 525/50 nm (green) emission filters^62^,^63^. A 450 nm signal indicates *M.tb* membrane disruption. As controls, non-infected THP-1 cells were treated without CCF-4 stain (gray) or with CCF-4 stain (green). (**d**) Median Fluorescence Intensity (MFI) of BV421, n = 3. *p < 0.05, one-way ANOVA and Tukey’s post test.

Given EsxA and other ESX-1 secreted substrates are virulence factors and some of the most immunodominant proteins during infection we examined the number of recruited T cells, *in vivo* cytokine expression and gross pathology in animals infected with *M.tb* Erdman or *M.tb* DK9897. Mice were aerosol infected with an inoculum of *M.tb* Erdman (10 CFU) or *M.tb* DK9897 (100 CFU) that ensured that the bacteria number was approximately 100.000 per lung in both groups after six weeks infection (Supplementary Figure S4). At this time point, we also determined the total number of CD4 T cells in the lungs by flow cytometry (Supplementary Figure S4). In aged matched non-infected animals, we found 0.55 × 10^6^ +/− 0.27 CD4 T cells. This number increased 2.7-fold after *M.tb* Erdman infection (1.48 × 10^6^ +/− 0.65) and 2.4 fold (1.30 × 10^6^ +/− 0.36) after *M.tb* DK9897 infection. Thus, for both infections, the recruitment of approximately 60% of the lung CD4 T cells was due to the ongoing infection. Restimulation of the lung T cells with EsxH showed that approximately the same frequency of CD4 T cells could recognize and respond to the antigen *in vitro* (Supplementary Figure S1). However, when comparing *in vivo* expression of cytokines in the lungs of infected animals the results were quite different despite similar bacteria and CD4 T cell numbers (Fig. 5a). In lungs infected with *M.tb* DK9897, there was almost no cytokine production *in vivo* whereas five of the nine cytokines tested were expressed in *M.tb* DK9417 infected lungs. These included the pro-inflammatory cytokines IL-17, IL-6, IL-1β and TNF-α. In accordance, the expression of the anti-inflammatory cytokine IL-10 was neglectable in both infection groups. TNF-α is a Th1 cytokine often co-expressed in T cells with IFN-γ. Since we found strong expression of the latter in *M.tb* DK9417-infected lungs and no Th2 helper cell produced Interleukin 5 (IL-5) it suggests that there is a strong Th1/Th17 biased T cell response in *M.tb* DK9417-infected lungs that is almost absent in *M.tb* DK9897 infected lungs. Combined with an ongoing pro-inflammatory response that stimulates activation of phagocytes and increases recruitment of additional mononuclear leukocytes into the site of infection it leads to the accumulation of cells around the foci of infected cells and the formation of granulomas. Photographs of infected lungs confirm that *M.tb* Erdman-infected lungs have many and large granulomas in the lung tissue whereas *M.tb* DK9897 infected lungs have few and small granulomas (Fig. 5b).

### *M.tb* DK9897 does not make contact from the phagosome to the cytosol

Using a flow cytometry based *in vitro* approach we investigated the ability of *M.tb* DK9897 to disrupt the phagosomal membrane. To identify the optimal ratio of target cell and mycobacteria for the assay we first titrated the MOI for THP-1 cells and *M.tb* Erdman and found five MOI’s and four days incubation to give the best signal (Supplementary Figure S5). Thus, THP-1 cells were infected at a MOI of five with either *M.tb* DK9897, *M.tb* Erdman or *M.bovis* BCG. Four days later the cytosol of THP-1 cells was loaded with CCF-4 dye. If the mycobacteria are capable of disrupting the phagosomal membrane bacterial expressed β-lactamase can translocate into the cytosol where it cleaves CCF-4 and causes a fluorescent shift from 535 nm to 450 nm. Four days after infection, we found a clear shift in fluorescent for the three independent *M.tb* Erdman-infected samples whereas there was no shift in the *M.bovis* BCG or *M.tb* DK9897 infected samples. Thus, *M.tb* Erdman can disrupt the phagosomal membrane whereas *M.tb* DK9897 cannot (Fig. 5c).

## Discussion

The mycobacterial ESX-1 secretion system was linked with increased pathogenicity and host-pathogen interactions soon after its discovery^36^,^37^. Our results are in line with the growing pool of evidence showing the importance of the ESX-1 secretion system for the virulence of *M.tb*^38^,^39^,^40^ and the generation of a host immune response exploited by the mycobacteria to increase growth and spreading^41^. Furthermore, our study shows that an attenuated *M.tb* strain lacking ESX-1 secretion can cause cervical tuberculous lymphadenitis. Although this is the first description of a clinical *M.tb* strain with a defect in ESX-1 secretion, there are cases of extra-pulmonary TB caused by *Mycobacterium microti* or *Mycobacterium bovis* BCG, both lacking ESX-1 function^42^,^43^. Most likely, these strains primarily cause disease in immune compromised individuals and since our case was a 92-year-old patient it is possible, she had developed one or more immune-deficiencies. Our MIRU analysis showed that *M.tb* DK9897 is a rare strain belonging to a mycobacterial cluster having very few members. However, mutations impairing EsxA secretion can occur independently in different phylogenetic lineages, we, therefore, searched the publically available *M.tb* complex genome sequences for mutations with major effect on the ORFs of ESX-1 related genes. To our surprise, we found 18% of the sequenced strains to have a mutation in one or more of the searched ESX-1 related genes. This is not in line with the importance of ESX-1 for virulence or that EsxA and EsxB based assays in numerous studies have diagnosed TB patients with sensitivities around 90%^44^. Most likely, the discrepancy is due to sequencing errors in the deposited genome sequences where several of the genomes have relatively low sequence coverage. Alternatively is ESX-1 secretion during a human infection not critically dependent on the same proteins that have been found to be important for ESX-1 secretion in mice.

**Figure 6:**
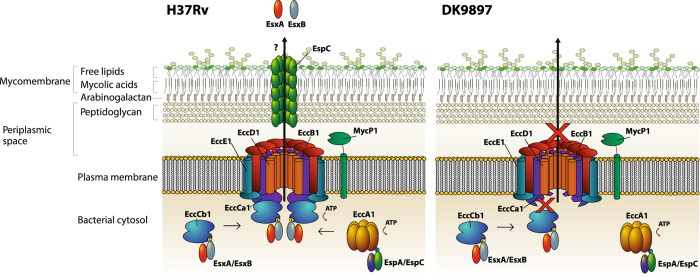
Model of the functional ESX-1 secretion apparatus and the effect of the modified *eccCa1* (Rv3870) in *M.tb* DK9897. (**a**) The core structure of the ESX-1 system is formed by the conserved components EccB1, EccCa1, EccCb1, EccD1 and EccE1, each possessing transmembrane region(s) spanning the mycobacterial plasma membrane^64^. The EccC is an ATP-driven translocase consisting of two subunits, EccCa1 and EccCb1, which are assembled once EccCb1 binds its target substrate, in this case, the EsxA/EsxB heterodimer, where EccCb1 interacts with the carboxy-terminal signal sequence of EsxB (marked as C in the figure)^65^,^66^. EsxB functions as a chaperone for EsxA secretion, which is a major ESX-1 virulence factor^21^. The secretion of EsxA/EsxB is co-dependent with the secretion of EspC/EspA and the C-terminus of EspC targets for the interaction with the cytosolic ATPase EccA1^67^,^68^. EspC polymerizes during secretion, indicating that EccA1 and EspA might function as cytosolic chaperones^69^. The polymerization of EspC results in the formation of a surface-exposed filamentous structure that spans the entire cell envelope^69^, possibly serving as a channel responsible for transporting ESX-1 substrates. (**b**) The truncated EccCa1 of *M.tb* DK9897 is unable to bind the EccCb1/EsxA/EsxB complex and is thus unable to secrete the EsxA/EsxB heterodimer. The secretion of EsxA/EsxB and EspA/EspC are mutually co-dependent^67^, meaning that the secretion of EspC is presumably also disrupted in *M.tb* DK9897.

For our virulence investigation, we used a murine TB model to compare the ability of *M.tb* DK9897 and *M.tb* Erdman to grow after an aerosol infection. The model assesses the overall ability of a strain to enter the lung, infect alveolar macrophages, survive and replicate within macrophages. The low CFU load after six weeks infection suggest an attenuation of *M.tb* DK9897. We found similar numbers of bacilli in the lungs of *M.tb* Erdman and *M.tb* DK9897 infected animals shortly after aerosol infection. Thus, attenuation is unlikely to be due to a defect in establishing the initial infection and because of the slower *in vitro* growth rate, it is likely that one or more of the approximately 1200 mutations in the *M.tb* DK9897 genome sequence has a major influence on the doubling time of exponential growing *M.tb* DK9897. Since we know that ESX proteins induce T-cell responses^45^, the lack of a T cell response against EsxA that was restored by gene complementation confirmed the connection between *in vivo* attenuation and secretion of EsxA. Western blot analysis of mycobacterial pellets and supernatants from *in vitro* grown cultures revealed that *M.tb* DK9897, unlike *M. bovis* BCG, was able to express but not secrete EsxA. EsxA is transported across the bacterial membrane in a heterogeneous dimer complex with EsxB by the ESX-1 system (Fig. 6). Previous studies have shown that several genes, encoded from at least two chromosomal loci, are required for export of EsxA^16^,^18^,^37^,^46^. In order to identify a potential defect in any of the genes previously identified as essential for ESX-1 secretion, we sequenced the *M.tb* DK9897 genome and compared it to the *M.tb* H37Rv database sequence^35^. The comparative analysis revealed around 1200 polymorphisms to the published sequence, among which a single nucleotide insertion in the *eccCa1* gene stood out as a probable explanation for the lack of ESX-1 activity. In virulent mycobacteria, *eccCa1* encodes a 747 amino acid long protein involved in ATP cleavage and generation of energy to move proteins across the bacterial membrane through the ESX-1 system^47^. The *eccCa1* insertion in *M.tb* DK9897 causes a frameshift, resulting in an EccCa1 protein lacking 305 amino acids at the C-terminus (Supplementary Figure S4). This is in agreement with an earlier study, showing attenuated growth of *M.tb* H37Rv in the human macrophage-like THP-1 cell line and in aerosol-infected mice and a lack of EsxA and EsxB secretion when the *eccCa1* gene was disrupted^16^. Given the central function of EccCa1 in ESX-1 secretion, it is likely that ESX-1 does not secrete any proteins in *M.tb* DK9897 (Fig. 6). To confirm this hypothesis, we attempted to identify lack of another ESX-1 secreted protein, EspC, in the supernatant of *M.tb* DK9897 cultures using two polyclonal antibody preparations. Unfortunately, we were unable to get a signal using this approach, not even in positive control samples from fully virulent *M.tb* Erdman cultures.

To confirm that the defect in ESX-1 was due to the nucleotide insertion in the *eccCa1* gene, we complemented *M.tb* DK9897 with a plasmid-borne version of the *M.tb* H37Rv *eccCa1-eccCb1-pe35* genes^16^. This complementation restored secretion of, and T cell responses against, EsxA and the virulence of the recombinant mycobacteria increased slightly. This was quite similar to the modest increase in virulence that occurred after knock-in of the RD1 region into *M.bovis* BCG^15^. Thus, attenuation of *M.tb* DK9897 is partly due to a single nucleotide insertion in the *eccCa1* gene disrupting the encoded proteins ATPase activity. This eliminates ESX-1 secretion of virulence factors and they become non-functional in bacterial survival and spreading. We did also identify a mutation in the *pe35* gene from DK9897 compared to the *M.tb* H37Rv reference that potentially could be the culprit for the secretion defect (Supplementary Table S2). However, we find this unlikely because the mutation only affects the last amino acid in PE35, changing a glutamate codon into a STOP codon resulting in the expression of a PE35 protein lacking one amino acid at the C-terminal end. Furthermore, searching the NCBI database, we found that 83% (117 out of 141) of the sequenced genomes from the *M.tb* complex have exactly the same *pe35* gene sequence as *M.tb* DK9897, the rest have the *M.tb* H37Rv sequence. It is possible that part of the EccCa1 associated attenuation could be due to an impairment of the cell wall integrity but we find this unlikely since we could not identify the truncated EccCa1 protein in *M.tb* DK9897 cultures^48^. Since complemented *M.tb* DK9897 is not as virulent as *M.tb* H37Rv, it is likely that one or more of the other sequence changes in *M.tb* DK9897 contributes to the strain attenuation via a different pathway that has an influence on both *in vivo* and *in vitro* growth.

To compare the host immune responses against *M.tb* Erdman and *M.tb* DK9897 we infected mice with different inoculums of the strains to allow them to reach the same CFU levels after six weeks infection. At this time point, there were approximately three-fold more T cells in *M.tb* Erdman-infected lungs than non-infected lungs and slightly fewer cells in *M.tb* DK9897 infected lungs. When lung CD4 T cells were stimulated *in vitro*, in the presences of excess antigen and co-stimulatory molecules, they were capable of responding and producing cytokines regardless of the challenge strain. However, *in vivo*, there was a big difference in cytokine expression. In mice infected with virulent *M.tb*, there was an expression of both pro-inflammatory and T cell cytokines whereas in *M.tb* DK9897 infected mice there was little or no cytokine production at all. Taken together, the *in vitro* and *in vivo* data suggest that there is limited antigen presentation and T cell activation in the chronic stage of an *M.tb* DK9897 infection, while the virulent strain induced an increased and more persistent response. The prolonged induction of immune responses leads to tissue destruction and, in accordance, we found many and large granulomas in lungs from mice infected with virulent *M.tb* whereas there was almost no gross pathology in lungs from *M.tb* DK9897 infected mice. Granuloma formation has been associated with *M.tb* escaping from the phagosome to the cytosol and induction of host cell death via necrosis^39^,^49^. We, therefore, investigated the difference in the two strains’ ability to disrupt the phagosomal membrane and make a contact to the cell cytosol. *M.tb* Erdman clearly can establish this contact, whereas *M.tb* DK9897 and *M. bovis* BCG is incapable of this. Similar results were found in a previous study where pathogenic *M.tb* and *Mycobacterium leprae* made contact to the cytosol of the host cell and induced cell death whereas non-pathogenic *M. bovis* BCG did not^50^.

Thus, our data are in agreement with a model where virulent *M.tb* exploits ESX-1 to disrupt the phagosomal membrane and escapes into a more permissive environment, followed by a rapid expansion of *M.tb* and increased cell-to-cell spreading. The expansion and accumulation of high numbers of *M.tb* lead to host cell death, attraction, and activation of immune cells. Newly recruited immune cells become target cells for infection with free *M.tb*, while others, e.g. neutrophils, release cytokines and chemokines, thus increasing granuloma formation, pathology and further recruitment of cells. Faster replicating *M.tb* strains will induce more necrosis and accelerate the process^51^. In a human granuloma, there are few bacteria compared to a mouse granuloma and vacuolar-escaping is most likely a rare and transient event. However, it is still a critical step in bacterial expansion and cell-to-cell spreading, which we should understand in more detail to eliminate TB.

## Materials and Methods

### Animals

Six to eight week old female CB6F1 mice (Envigo, Scandinavia or Charles River, US) were rested for one week prior to initiation of experiments. Mice were housed under specific pathogen–free conditions in animal facilities at Statens Serum Institut (Copenhagen, DK) or the Center for Infectious Disease Research (Seattle, WA, US), provided with radiation-sterilized food and water *ad libitum*. Infected animals were housed in biosafety level 3 (BSL-3) facilities. The handling of mice was conducted in accordance with the regulations set forward by the respective national animal protection committees and in compliance with European Community Directive 2010/63 and the U.S. Association for Laboratory Animal Care recommendations for the care and use of laboratory animals. In agreement with the Danish Animal Welfare Act all experimental protocols involving animals were reviewed prior to the start of the experiment by an independent ethical review board at Statens Serum Institut and approved to be in accordance with our license for animal experiments issued by The Animal Experiments Inspectorate (License no 2014-15-2934-01065 and 2012-15-2934-00272) under the Ministry of Environment and Food of Denmark.

### Vaccines and Immunization

Animals were immunized subcutaneously (s.c.) at the base of the tail either once with 5 × 10^6^ CFU of BCG (BCG Danish 1331 (SSI, DK)) or three times at two-week intervals with 5 μg H56 in Cationic Adjuvant Formulation 01 (CAF01) (SSI, DK)^30^. H56 was recombinantly produced in *E. coli*.

### Mycobacterial strains and culture conditions

The *M.tb* clinical strains, DK9897, and *M.tb* Beijing strain, DK9417, were obtained from the *M.tb* strain collection at the International Reference Laboratory of Mycobacteriology, Statens Serum Institut, Copenhagen, Denmark. *M.tb* Erdman and *M.tb* H37Rv were obtained from the American Type Culture Collection (ATCC 27294). All *M.tb* strains used in this study were grown at 37 °C for 3–4 weeks either on solid medium (Middlebrook 7H11) or in liquid medium (Middlebrook 7H9) supplemented with 10% (v/v) oleic acid–albumin–dextrose–catalase (OADC). For the *in vitro* growth curves, the 7H9 with 10% OADC was supplemented with 0.2% (v/v) glycerol and 0.025% (v/v) Tween 80. The handling of strains was done in BSL-3 facilities at either Statens Serum Institut, Denmark or the Center for Infectious Disease Research, WA, USA.

### Mycobacterial challenge

Six weeks after the third immunization animals were infected with 100 viable bacilli by the aerosol route^30^, with *M.tb* Erdman, *M.tb* DK9417, *M.tb* DK9897 or *M.tb* DK9897 complemented with pKG18 (DK9897::pKG18)^16^. Six weeks after infection, mice were sacrificed and organs homogenized in PBS for bacterial enumeration, as described in^30^, and flow cytometry analysis.

### Genotyping, Sequencing and Phylogenetic analysis

MIRU-VNTR 24-locus genotyping and DNA extraction from bacterial cultures were performed as previously described^52^. A minimum spanning tree was created with the MIRU-VNTRplus database website. MIRU-VNTR clusters were defined as strains with identical genotyping patterns and clonal complexes by a maximum difference of two loci^53^. Genomic DNA of *M.tb* DK9897 was sequenced, mapped and variance analyzed by Eurofins, Ebersberg, Germany. Sequencing was done using the MiSeq system (Illumina, San Diego, California, USA) with 300 base pair (bp) paired-end reads from a Nextera DNA library, yielding a total of 2359 Mbp calls. Low-quality bases (Phred quality score <20) and clipped reads shorter than 40 bp were removed using Trimmomatic (v0.32)^54^ using the sliding window approach (size 4 bp) resulting in a mean read length of 225 bp. The trimmed reads were mapped to the *M.tb* strain H37Rv genome (GenBank ID: NC_000962.3) using the Burrows–Wheeler Aligner BACKTRACK (v0.6.2-r126)^55^ resulting in a mean genome coverage of 365. Variant analysis was performed using VarScan (v2.3)^56^ aligning reads with a quality of at least 1 (Phred quality score). The variants were classified into categories: LOW (synonymous variant), MODERATE (missense variant, in frame insertion/deletion) and HIGH (stop lost/gained, frameshift insertion/deletion). Results were analyzed using Excel (v15.0.4885.1000). The complete annotation of the *M.tb* DK9897 genome is located under GenBank accession no. CP018778.

### Alignment of ESX-1 associated genes from public available genomes

The presence and integrity of twelve ESX-1 related genes were analyzed in a panel of *M.tb* complex strains and clinical isolates for which the genomes had been completely sequenced. These organisms were selected from the Nucleotide collection database of the National Center for Biotechnology Information (NCBI), limiting the search by organism—Mycobacterium tuberculosis complex (taxid: 77643). The search was performed on the 7th of March 2017 using the nucleotide sequences for twelve genes from the reference strain *M.tb* H37Rv as input (espC, espA, ecca1, eccB1, eccCa1, eccCb1, pe35, esxA, esxB, eccD1, eccE1, and mycP1) and the BLASTN (v2.6.1+) program^57^. Homologous genes were identified and individually aligned against their reference orthologue from *M.tb* H37Rv to identify nucleotide substitutions, insertions, and deletions.

### Lymphocyte cultures, ELISA/MSD, and flow cytometry

Lymphocytes were isolated from spleens and lungs as described previously^58^. Cultures were adjusted to 2 × 10^5^ cells/well for MSD/ELISA or 1–2 × 10^6^ cells/well in a total volume of 200 μl/well for flow cytometry and stimulated with antigens at a final concentration of 2 μg/ml. Concanavalin A was used at a concentration of 1 μg/ml as a positive control for cell viability. Culture supernatants were harvested after 72 h of incubation for the investigation of IL-17 ELISA. Lymphocytes for IC-FACS were stimulated at 37 °C in the presence of recombinant antigen (2 μg/ml) for 1 hour, and subsequently incubated for 6 hours after adding 10 μg/ml Brefeldin A (Sigma-Aldrich) and stained^59^. The following antibodies were used for surface staining: anti-CD4-APC (clone RMA4-5, BD; 1:600), anti-CD44-FITC (clone IM7, eBioscience; 1:600). For intracellular staining, the following antibodies were applied in 1:200 dilutions: anti-IL-17A-PerCp-Cy5.5 (clone eBio17B7; eBiosciences), anti-IFN-γ-PE-Cy7 (clone XMG1.2; eBiosciences), anti-TNF-α-PE (MP6-XT22; eBiosciences), anti-IL-2-APC-Cy7 (clone JES6-5h4; BD). Responses were analyzed using a FACSCanto or LSRFortessa flow cytometer (BD) and FlowJo v.10.2 (Tree Star Inc.).

### Multiplex cytokine assay

The Meso Scale Discovery multiplex mouse cytokine assay was used (IFN-γ, IL-1β, IL-2, IL-6, IL-5, IL-10, TNF-α, IL-12) and performed according to the manufacturer’s instructions. The plates were read on the Sector Imager 2400 system (Meso Scale Discovery) and calculation of cytokine concentrations in unknown samples was determined by 4-parameter logistic non-linear regression analysis of the standard curve.

### SDS-PAGE and Western blot analysis

Preparation of mycobacterial protein extracts and culture filtrates was performed as described elsewhere^60^,^61^. 4–20% Mini-PROTEAN^®^ TGX precast gels (Bio-Rad, Hercules, CA) were used, and 20 μg of concentrated culture filtrates of *M.tb* Erdman, H37Rv, DK9897 or DK9897::pKG18 or 2.5 μg disrupted bacterial pellet from the corresponding samples were applied in each lane. Proteins were transferred to nitrocellulose by the Trans-Blot^®^ Turbo™ transfer system (BioRad), the nitrocellulose was blocked in PBS with 5% skimmed milk, 0.1% Tween 20 and incubated with either anti-EsxA (HYB76-8) or anti-Ag85B (TD17) murine antibodies diluted 1:500. Antigen-specific immunoglobulins were detected with alkaline phosphatase-conjugated secondary antibodies (DAKO, Agilent, DK) or using enhanced chemiluminescent with HRP conjugated secondary antibodies (SuperSignal West Pico, Thermofischer).

### Cell cultures and infection

THP-1 cells (ATCC) were maintained in RPMI supplemented with 10% heat-inactivated focal calf serum (FCS). THP-1 cells were seeded in 12-well plates at a concentration of 0.2 × 10^6^ cells/ml and treated with 20 ng/ml of Phorbol 12-Myristate 13-Acetate (PMA) for 72 h to induce their differentiation into monocyte-derived macrophages.

THP-1 derived macrophages were infected at various multiplicity of infection (MOI) of *M.tb* Erdman, *M.tb* DK9897 or BCG in antibiotic free complete RPMI for 2 h at 37 °C, 5% CO_2_. The infectious medium was removed and cells were complemented with fresh growth medium containing 10% FCS. At time course measurements cells were harvested with a cell scraper and moved to a 96 well plate for analysis.

### CCF-4 assay and flow cytometry

Cells were stained with a 1X final CCF4-AM solution (Life Technologies) in PBS according to the manufacturer’s instructions for 2 h at room temperature (RT). Cells were washed three times with PBS and then stained with Live/dead reagent (APC-eF780, 1:1000) and fixed with 4% paraformaldehyde (PFA). Cells were analyzed in a BD LSRFortessa™ using BD FACSDIVA™ software (BD Bioscience). Data analysis were done with FlowJo v10.2 software. CCF-4 fluorescence was measured using 405 nm as excitation laser and 450/50 nm (blue) and 525/50 nm (green) emission filters (as illustrated in^62^).

### Statistical analysis

Prism 7 software (GraphPad v7.02) was used for all statistical analyses. Bacterial numbers were log-transformed before being analyzed using one-way ANOVA with Tukey’s multiple comparisons test. *In vivo* cytokine levels were analyzed using two-tailed t-tests with Mann-Whitney correct. Statistically significant differences are marked by asterisks in figures and explained in the figure legends.

## Additional Information

**How to cite this article**: Clemmensen, H. S. *et al*. An attenuated *Mycobacterium tuberculosis* clinical strain with a defect in ESX-1 secretion induces minimal host immune responses and pathology. *Sci. Rep.* **7**, 46666; doi: 10.1038/srep46666 (2017).

**Publisher's note:** Springer Nature remains neutral with regard to jurisdictional claims in published maps and institutional affiliations.

## Supplementary Material

Supplementary Data

## Figures and Tables

**Table 1:** Summary of identified mutations in the *M.tb* DK9897 strain relative to the H37Rv genome.

	DK9897
Missense variant	628
Synonymous variant	471
Stop gained	13
Stop lost	1
Frameshift variant (Ins + Del)	54
Inframe insertion	10
Inframe deletion	11
Disruptive inframe deletion	9
Disruptive inframe insertion	2
Total	1199

Missense variant: a sequence variant that changes one or more bases, resulting in a different amino acid sequence but where the length is preserved.

Synonymous variant: a sequence variant where there is no resulting change to the encoded amino acid.

Stop gained: a sequence variant whereby at least one base of a codon is changed, resulting in a premature stop codon, leading to a shortened cds (codon DNA sequence).

Stop lost: a sequence variant where at least one base of the terminator codon (stop) is changed, resulting in an elongated cds.

Frameshift variant (Ins + Del): a sequence variant which causes a disruption of the translational reading frame, because the number of nucleotides inserted or deleted is not a multiple of three.

In frame insertion: an in frame non-synonymous variant that inserts bases into in the coding sequence.

In frame deletion: an in frame non-synonymous variant that deletes bases from the coding sequence.

Disruptive in frame deletion: an in frame decrease in cds length that deletes bases from the coding sequence starting within an existing codon.

*M.tb* H37Rv: NC_000962.

Mutations were classified based on their potential effect on protein function/expression: High; stop gained, stop lost and frameshift variants. Moderate; missense mutant, in frame insertion, in frame deletion, disruptive in frame deletion, disruptive in frame insertion, synonymous variant. Low; synonymous variant.
