# Supplementary material for: An attenuated Mycobacterium tuberculosis clinical strain with a defect in ESX-1 secretion induces minimal host immune responses and pathology
Source: Sci Rep. 2017 Apr 24;7:46666. doi: 10.1038/srep46666 (PMC5402389; doi:10.1038/srep46666)
Supplement: Supplementary Data [file srep46666-s1.doc]

**Supplementary data for:**

**An attenuated *Mycobacterium tuberculosis* clinical strain with a defect in ESX-1 secretion induces minimal host immune responses and pathology.**

**Helena Strand Clemmensen*1, Niels Peter Hell Knudsen*1, Erik Michael Rasmussen2, Jessica Winkler3, Ida Rosenkrands1, Ahmad Ahmad1, Troels Lillebaek2, David R. Sherman3, Peter** **Lawætz Andersen1, Claus Aagaard#1**

**1: Department of Infectious Disease Immunology, Statens Serum Institut, DK-2300 Copenhagen, Denmark**

**2: International Reference Laboratory of Mycobacteriology, Statens Serum Institut, DK-2300 Copenhagen, Denmark**

**3: Center for Infectious Disease Research, University of Washington, Seattle, Washington 98109, USA**

***Co-first author**

**# Correspondence caa@ssi.dk**

**Supplementary table S1. MIRU analysis.** The 24-locus MIRU-VNTR analysis of *M.tb* DK9897 compared to the related *M.tb* isolates Erdman, H37Rv and DK9417 commonly used in TB research. The table indicates the number of tandem repeats at each of the 24 tested loci.


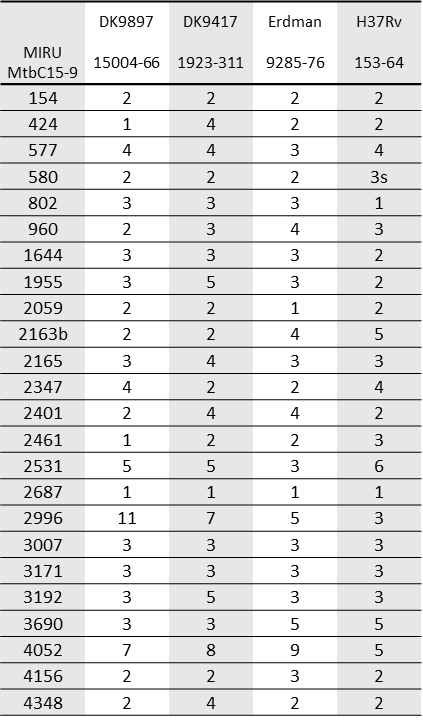


**Supplementary table S2. Major *M.tb* DK9897 genome polymorphisms**. Genome changes in *M.tb* DK9897 ORFs - relative to the *M.tb* H37Rv genome sequence. Fs = frame shift; * stop codon

**
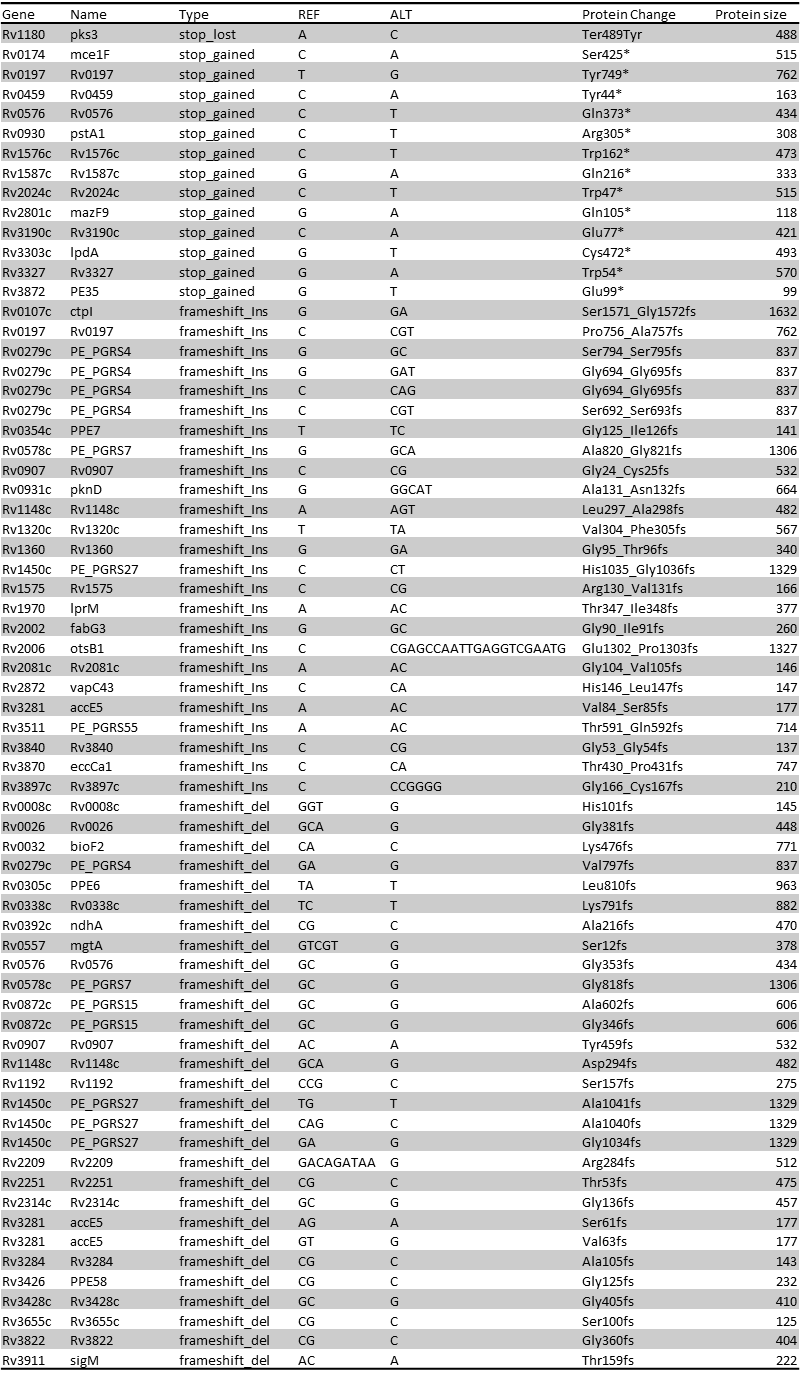
**

**Supplementary table S3. ESX-1 gene polymorphisms in public available genomes.** Clinical isolates with alternative sequences relative to the orthologous *M.tb* H37Rv sequences. X marks isolates with either a frameshift or gain/loss of a stop codon in the respective gene. The coverage indicates the average depth of coverage during sequencing of the genome.

| **Name** | **EspC** | **EspA** | **EccA1** | **EccB1** | **EccCa1** | **EccCb1** | **PE35** | **EsxA** | **EsxB** | **EccD1** | **EccE1** | **MycP1** | **Type** | **Strain** | **Coverage** |
| --- | --- | --- | --- | --- | --- | --- | --- | --- | --- | --- | --- | --- | --- | --- | --- |
| CP008971 | x | x |  |  |  | x |  | x |  |  |  |  | Mycobacterium tuberculosis | 0B049XDR | 25 |
| CP003234 | x |  | x |  | x | x |  |  |  | x | x |  | Mycobacterium tuberculosis | RGTB423 | 25 |
| AP017901 |  | x |  |  |  |  |  |  |  |  |  |  | Mycobacterium tuberculosis | NCGM946K2 | 773 |
| CP005386 |  | x | x | x |  |  |  |  |  |  | x | x | Mycobacterium tuberculosis | CAS/NITR204 | 60 |
| CP010335 |  | x |  |  |  | x |  |  |  |  |  |  | Mycobacterium tuberculosis | 2242 | 89 |
| CP010334 |  | x |  |  |  |  |  | x |  | x |  |  | Mycobacterium africanum | 25 | 95 |
| CP005387 |  | x |  |  |  |  |  |  |  |  |  |  | Mycobacterium tuberculosis | EAI5/NITR206 | 60 |
| CP008969 |  |  | x |  |  |  |  | x |  |  |  |  | Mycobacterium tuberculosis | 0B076XDR | 25 |
| CP008967 |  |  |  | x |  |  |  |  |  |  |  | x | Mycobacterium tuberculosis | 0B169XDR | 25 |
| CP010332 |  |  |  | x |  |  |  |  |  |  |  |  | Mycobacterium bovis | 30 | 94 |
| CP001641 |  |  |  | x |  |  |  |  |  |  |  |  | Mycobacterium tuberculosis | CCDC5079 | 10 |
| CP003233 |  |  |  | x |  | x |  |  | x | x | x |  | Mycobacterium tuberculosis | RGTB327 | 25 |
| CP008972 |  |  |  | x |  | x |  |  |  |  | x |  | Mycobacterium tuberculosis | 0B026XDR | 25 |
| CP018778 |  |  |  |  | x |  |  |  |  |  |  |  | Mycobacterium tuberculosis | DK9897 | 365 |
| CP008980 |  |  |  |  |  | x |  |  |  |  |  |  | Mycobacterium tuberculosis | 0A033DS | 25 |
| CP008979 |  |  |  |  |  | x |  |  |  |  |  |  | Mycobacterium tuberculosis | 0A036DS | 25 |
| CP008964 |  |  |  |  |  | x |  |  |  |  |  |  | Mycobacterium tuberculosis | 0B228DS | 25 |
| CP008966 |  |  |  |  |  | x |  |  |  |  |  |  | Mycobacterium tuberculosis | 0B218DS | 25 |
| CP010339 |  |  |  |  |  |  | x |  |  |  |  |  | Mycobacterium tuberculosis | 22103 | 101 |
| CP010329 |  |  |  |  |  |  |  |  |  | x |  |  | Mycobacterium tuberculosis | F1 | 120 |
| CP008974 |  |  |  |  |  |  |  |  |  | x |  | x | Mycobacterium tuberculosis | 0A115DS | 25 |
| CP010336 |  |  |  |  |  |  |  |  |  | x |  |  | Mycobacterium tuberculosis | 2279 | 97 |
| CP008963 |  |  |  |  |  |  |  |  |  | x |  |  | Mycobacterium tuberculosis | 0B229DS | 25 |
| CP008983 |  |  |  |  |  |  |  |  |  | x |  |  | Mycobacterium tuberculosis | 0A005DS | 25 |
| CP008975 |  |  |  |  |  |  |  |  |  |  | x |  | Mycobacterium tuberculosis | 0A094DS | 25 |
| CP009427 |  |  |  |  |  |  |  |  |  |  |  | x | Mycobacterium tuberculosis | 96121 | 25 |
|  |  |  |  |  |  |  |  |  |  |  |  |  | **Number of genomes** | 26 |  |
|  |  |  |  |  |  |  |  |  |  |  |  |  | **Percentage of total** | 18% |  |
|  |  |  |  |  |  |  |  |  |  |  |  |  |  |  |  |

**Supplementary Figure S1. Frequency of cytokine producing CD4 T cells after antigen stimulation.** Lung cells isolated from vaccinated and non-vaccinated mice infected for six weeks with either *M.tb* Erdman or *M.tb* DK9897 where incubated for six hours with 2 µg/ml of the indicated proteins where after cytokine production was measured in individual cells by flow cytometry.

**Supplementary Figure S2. Full length western blots.** A) The full length blots of the cropped blots shown in Figure 2C. B) The full length blots of the cropped blots shown in Figure 4A.


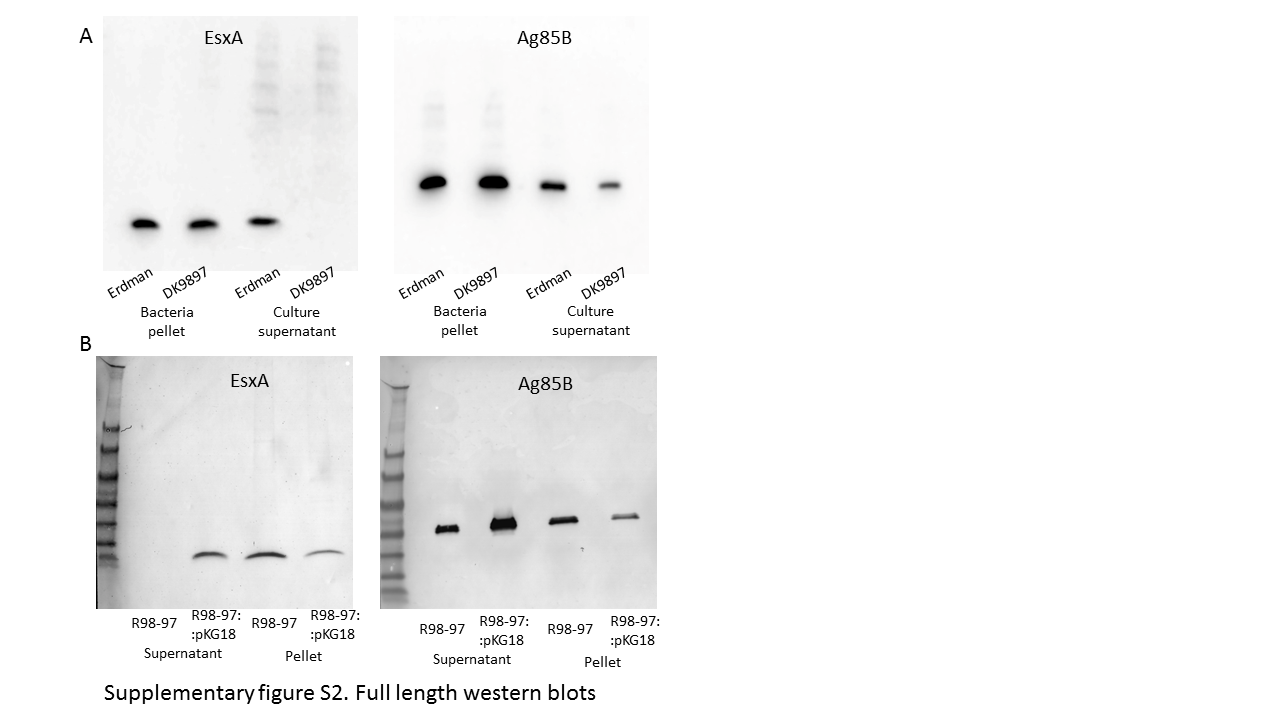


**Supplementary Figure S3**. **Amino acid sequence of the potentially truncated EccCa1 (Rv3870) and predicted transmembrane regions**. The peptide sequences used to raise antibodies for identification of the shortened protein by western blot is marked in blue. The putative twelve amino acid sequence added to the EccCa1 sequence because of the frame shift is marked in red.

**X** indicates the induced stop codon in *M.tb* DK9897 EccCa1.

**>*M. tb DK9897,* truncated EccCa1**

MTTKKFTPTITRGPRLTPGEISLTPPDDLGIDIPPSGVQKILPYVMGGAMLGMIAIMVAGGTRQLSPYMLMMPLMMIVMMVGGLAGSTGGGGKKVPEINADRKEYLRYLAGLRTRVTSSATSQVAFFSYHAPHPEDLLSIVGTQRQWSRPANADFYAATRIGIGDQPAVDRLLKPAVGGELAAASAAPQPFLEPVSHMWVVKFLRTHGLIHDCPKLLQLRTFPTIAIGGDLAGAAGLMTAMICHL**AVFHPPDLLQIRVLTEEPDDPDWSWLKWLPHVQHQTETDAAGSTRLIFTRQEGLSDLAARGPHAPDSLPGGP**Y**VVVVDLTGGKAGFPPDGRAGVTVITLG**NHRGSAYRIRVHEDGTADDRLPNQSFRQVTSVTDRMSPQQASRIARKLAGWSITGTILD**KTSRVQKKVATDWHQ**LVGAQSVEEITTFPLEDVHRHRP*

Theoretical pI/Mw: 8.98 / 47861.15


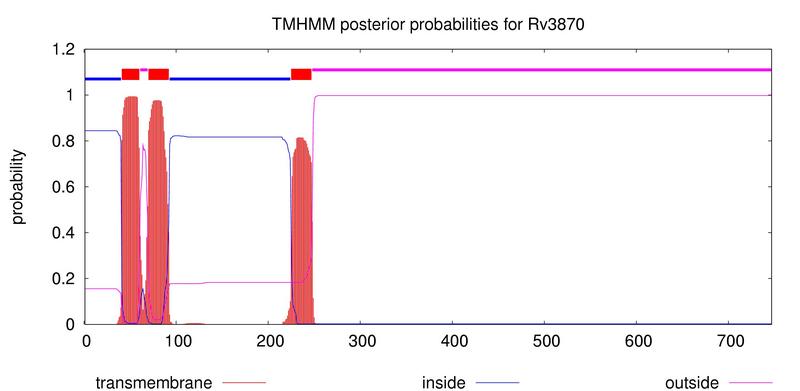


**Supplementary Figure S4. Bacteria and CD4 T-cell numbers in lungs.** **A**) Number of *M.tb* bacteria in lungs from mice infected with either *M.tb* Erdman or *M.tb* DK9897 for six weeks. N=6 lungs for each infection. Mean and SEM´s of log transformed bacterial numbers are indicated. The inoculum size was adjusted to give similar CFU´s after six weeks infection. Approximately 10 CFU of *M.tb* Erdman and 100 CFU of *M.tb* DK9897 delivered into the lungs of individual mice. **B**) Number of CD4 T-cells in the same lungs.

A B

**Supplementary Figure S5**. **Phagosome escape correlates with higher MOI of *M.tb* Erdman**. THP-1 cells were differentiated with PMA for 72h and infected with 1, 2.5, 5 or 10 MOI of *M.tb* Erdman for 2h. Cells were stained 3h post infection with CCF-4 for 2h at RT and analyzed with flow cytometry. NI: non-infected.


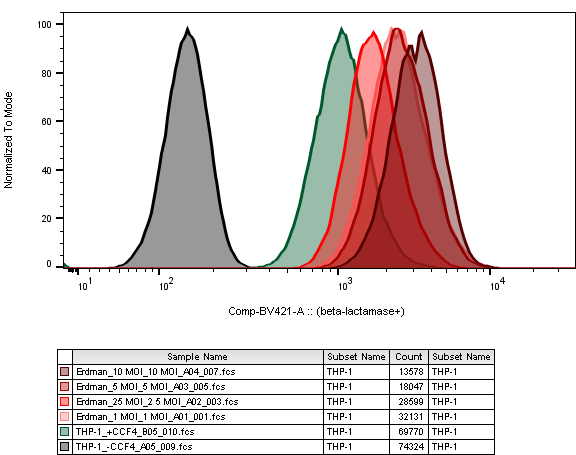

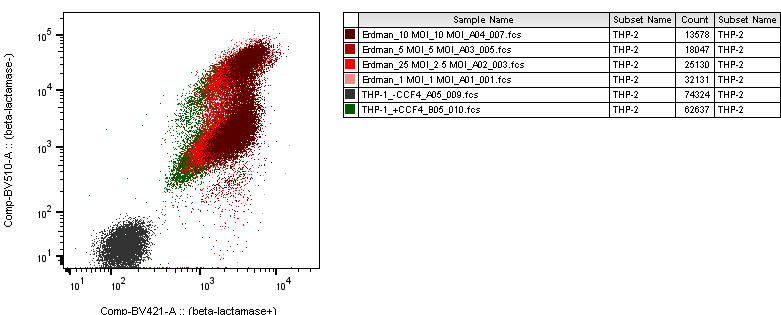


Cell counts

10 MOI

5 MOI

2,5 MOI

NI w/o CCF4

NI w CCF4

1 MOI

Bv421 (β-lactamase+)

Bv421 (β-lactamase+)
